# A symmetry analysis methodology for general energy conversion systems

**DOI:** 10.1038/s44172-023-00096-x

**Published:** 2023-07-25

**Authors:** Huan Guo, Yujie Xu, Yifu Li, Lujing Huang, Haisheng Chen

**Affiliations:** 1 Institute of Engineering Thermophysics, Chinese Academy of Sciences, 11 Beisihuanxi Rd, Haidian District Beijing, 100190 China; 2 University of Chinese Academy of Sciences, No.19(A) Yuquan Rd, Shijingshan District Beijing, 100049 China; 3 Department of Mechanical and Mechatronics Engineering, University of Waterloo, 200 University Avenue West, Waterloo, ON N2L 3G1 Canada

**Keywords:** Energy modelling, Power stations

## Abstract

Symmetry is a useful concept that has guided many scientific developments in fields such as structural engineering, data, and materials science. Here we apply a symmetry analysis method to explore the relationship between symmetry, output work and efficiency in macroscopic energy conversion systems. Brayton cycle is used as an example. A specific potential-displacement-energy (PDE) diagram was established for system symmetry analysis. Results prove that the symmetry of thermodynamic cycles could predict the output work and the efficiency. Stronger rotational symmetry generates more work while reflection symmetry leads to higher efficiency at constant specific heat capacity (*c*_p_). The condition for varied *c*_p_ to keep intermediate maximum-work temperature constant is greatly broaden. A more symmetrical cycle with higher efficiency and larger output work is designed based on the symmetry analysis results. The results could also be applied to other thermodynamic cycles, such as Carnot cycle, which provides insights to design more efficient energy conversion cycles.

## Abstract (web summary)

Huan Guo and coworkers explore the relationship between symmetry, work and efficiency for macroscopic thermodynamic cycles. The results provide insights to design more efficient energy conversion cycles by enhancing the cycle symmetry.

## Introduction

Symmetry is a fascinating and ubiquitous natural phenomenon that inspires many innovative scientific developments^1–3^. For example, the group theory in mathematics, Noether theory in physics (each symmetry of an action quantity corresponds to a conservation law and owns a conservation quantity^4^), as well as the developments in microcosm researches (e.g., crystal study and quantum mechanical study^5^). In short, the concept of symmetry has guided studies of geometric structures, abstract data, patterns, and materials.

Although the symmetry concept is seldom used to guide macroscopic research, symmetry could still be potentially applied in these systems. Take the energy conversion system as an example, the input and output energies of a system lays the basics for the symmetry concept. Specifically, the symmetry of compressed air energy storage (CAES) systems has been identified in our previous work^6^. In CAES systems, the energy storage process converts electric energy into pressure energy and heat energy of air for storage, which are then converted back to electric energy in the energy release process. The energy release process is the inverse process of the energy storage process, thus these two processes are reflective symmetry. Theoretically, the more symmetrical the two processes are, the better the system performance is. In practical, since heat transfer requires temperature difference and air flow requires pressure difference, the energy storage process and the energy release process is not completely symmetrical. Thus, the degrees of the symmetry of these two processes represents the power of the system^7^. In addition, we applied the same concept and found symmetry in many other energy storage systems, including thermal storage system, flywheel, and heat pump electricity storage^6,8^.

One step further, we can generalize this symmetry concept to general thermodynamic cycles. In a thermodynamic cycle, the system always reaches a certain thermodynamic point from a starting point, and then returns to its origin to realize the transformation of energies^9^. Because the departure path and return path are often different, a thermodynamic cycle can be characterized as strong rotational symmetry but weaker reflection symmetry. Here, reflection symmetry means that there is a reflection symmetric relationship between two thermodynamic paths in the thermodynamic cycle, and the two thermodynamic paths are in opposite directions. It is defined that the closer the corresponding parameter points in these two processes are, the stronger the reflection symmetry of the thermodynamic cycle is. Rotational symmetry refers to the fact that after a thermodynamic cycle rotating at a certain angle on a thermodynamic diagram (such as the *T*-*s* diagram), the new thermodynamic-cycle diagram trends to be close to the original thermodynamic-cycle diagram. The higher the degree of similarity, the stronger the rotational symmetry of the cycle. In this study, we rationally propose the following two conjectures:

1. The rotational symmetry of general thermodynamic cycles can predict the quantity of output work (e.g., the strongest rotational symmetry corresponds to the maximum output work) under a certain analysis framework.
2. The reflection symmetry can predict the efficiency under the same analysis framework.

The above conjectures are based on a common practice to convert a thermodynamic cycle into a geometric pattern in a specific diagram (e.g., thermodynamic cycle in the *T*-*s* diagram). Therefore, the following questions need to be addressed in order to confirm our conjectures. (1) Whether the area enclosed by the transformed geometric pattern represents the work? (2) How does the rotational and reflection symmetry of the transformed geometric pattern reflect work and efficiency, respectively? In addition, the objective of this study is to establish an analytical method to reveal the relationship between symmetry, work output, and efficiency of a general thermodynamic cycle.

As a demonstration, this paper takes Brayton cycle as an example to establish a novel symmetry analysis method to explore the relationships between symmetry, work and efficiency. As one of the simplest but yet important thermodynamic cycles, Brayton cycle has been studied deeply in several studies^10–14^. For example, ref. ^10^ established the outstanding role of *η*_CA_ (Curzon–Ahlborn efficiency) as a superior limit for MW (maximum-work)-JB (Joule-Brayton) efficiencies in the case that thermal efficiencies depends on working fluid properties. The MW condition for JB cycles performing with working fluids that have constant *C*_p_ or *C*_p_ = *αT*^*n*^ is fulfilled by fluids with *C*_p_ = *a* + *bT*. Ref. ^12^ presented an analysis of thermal efficiency and its bounds at maximum power for thermal engines and the heat transferring processes are described by Newton’s law of cooling. In the short contact time limit, they recovered the famous CA efficiency in the first-order calculation. When they proceed to the second-order calculation, they derived a different efficiency formula and recovered the efficiency bounds at maximum power given by ref. ^15^. J Gonzalez-Ayala, et al.^11^ proposed a new connection between maximum-power Curzon–Ahlborn thermal cycles and maximum-work reversible cycles. This connection is built through a mapping between the exponents of a class of heat transfer laws and the exponents of a family of heat capacities depending on temperature. It was found that it is possible to use analytically closed expressions for maximum-work efficiencies to calculate good approaches to maximum-power efficiencies. However, it should be noted that the above studies obtained results through complex theoretical derivations and numerical calculations. To demonstrate the advantage of the proposed symmetry analysis method in this study, we are aimed to obtain the key characteristics and new insights of Brayton cycle (or similar thermal power conversion system) through the symmetry analysis method, which is a simpler approach compared to conventional method such as theoretical derivations and numerical calculations.

The major innovations of this work are summarized as follows: (1) The understandings of thermodynamic cycle and parameters are achieved from the perspective of symmetry, and more general parameter relationships are obtained; (2) A case study of symmetry analysis method for system optimal design is presented, which brings novel perspectives for general thermodynamic system optimal design; (3) The concept of potential and displacement in general energy conversion is revealed for creating new energy conversion cycles.

## Results and discussion

### The symmetry analysis for Brayton cycle

**Fig. 1:**
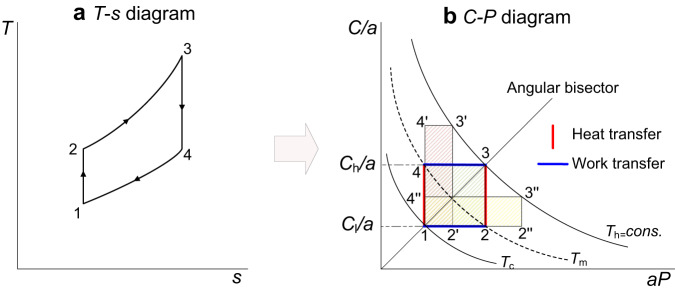
Conversion of *T*-*s* diagram to *C-P* diagram of Brayton cycle. **a** *T*-*s* diagram of Brayton cycle. Point 1 is the compressor inlet, point 2 is the heater inlet, point 3 is the expander inlet, and point 4 is the radiator inlet. **b** *C-P* diagram of Brayton cycle. *T*_h_ and *T*_c_ are the upper and lower temperature bounds of the cycle, respectively. *T*_m_ is the intermediate temperature. In the *C*-*P* diagram. The abscissa represents the pressure change in compressor/expander, which is related to work transfer. Therefore, the lateral movement of the point in the figure represents work transfer. For the similar reason, since the ordinate can reflect the temperature change of the working medium inside the heat exchanger, the longitudinal movement of the point in the figure represents heat transfer. The area enclosed by the rectangular cycle (shaded area) represent the net power output.

This section takes Brayton cycle as an example to demonstrate the proposed symmetry analysis method. Brayton cycle has two adiabatic processes (adiabatic compression process and adiabatic expansion process) and two isobaric processes (isobaric endothermic process and isobaric exothermic process) (see Fig. 1a). Ideal gas properties are considered in this section in order to introduce the symmetry method for easy understanding.

The entropy changes of a thermodynamic process from a starting state (state 1) to a terminal state (state 2) is calculated as

1 $$\varDelta {s}={{c}}_{{{{{{\rm{p}}}}}}}\,{{{{\mathrm{ln}}}}}({{T}}_{2}/{{T}}_{1})-{{R}}_{{{{{{\rm{g}}}}}}}\,{{{{\mathrm{ln}}}}}({{p}}_{2}/{{p}}_{1})$$

where *s* is entropy, *T* and *p* represent temperature and pressure, respectively, and *c*_p_ is specific heat at constant pressure and *R*_g_ is the gas constant.

In Eq. (1), it should be noted that state 2 can be any terminal state. Thus, the isotropic process of Δs = 0, *c*_p_ = *R*_g_*r*/(r–1), which is based on a specific-heat ratio *r* (*r* = 1.4 for diatom ideal gas) can be substituted into Eq. (1) to obtain:

2 $${T}/({{p}}^{(r-1)/r})={{T}}_{1}/({{{p}}_{1}}^{(r-1)/r})$$

As for the adiabatic process with increasing entropy, it be calculated by Eq. (3) which introduces the parameter *b*. The variable *C* obtained by this equation was defined as thermal-mechanical coefficient in our previous work^16^, which is further used in this study to evaluate the coordination degree between temperature and pressure. A proper *C* value can maintain the exergy efficiency of compressor and expander at a higher level. For example, for an expander whose outlet is an atmospheric environment, an appropriate coordination degree between temperature and pressure can enable the expander to convert the inlet energy of working medium into work to the maximum extent. If the coordination degree is not appropriate, the outlet temperature of the expander will deviate from the ambient temperature, resulting in a waste of exergy. The *C* value of a compressor is generally constrained by its inlet conditions. In order to achieve specific outlet temperature and pressure requirements, it is necessary to optimize the *C* value of the compressor to avoid energy waste at the compressor outlet. In addition, it should be noted that *b* and polytropic efficiency are reciprocal to each other (when *b* > 1) for the compression process, while *b* and polytropic efficiency are the same value (when *b* < 1) for the expansion process.

3 $${C}=\frac{{T}}{{{p}}^{({r}-1){b}/{r}}}$$

Based on the concept of *C*, a novel *C*-*P* diagram (*P* is the function of pressure, *P* = *p*^(*r-*1)*b/r*^) is developed, in which the *y*-axial is *C/a* and the x-axial is *aP*. *a* is a constant that adjusts the position of each thermodynamic point relative to the coordinate axes.

Figure 1 demonstrates the conversion of Brayton cycle from a conventional *T*-*s* diagram to the proposed *C*-*P* diagram (assuming the energy loss of compressor and expander is negligible, i.e., *b* = 1). Point 1 is the compressor inlet of the Brayton cycle, and its *C* and *P* values are calculated based on its thermodynamic parameters. The value of the constant *a* is derived to ensure that point 1 is on the angular bisector of *C*-*P* diagram. Then the position of point 1 on the *C*-*P* diagram is determined. It is worth noting that, the isotherm in the *C*-*P* diagram is a rectangular hyperbola curve (in the first quadrant) that is symmetric about the angular bisector line (see Fig. 1b) since the *x*-axial times the *y*-axial equals temperature *T* (see Eq. (3)). Therefore, compared to the *T*-*s* diagram, on the *C*-*P* diagram, the Brayton cycle can be expressed as a symmetrical rectangular structure, and the temperature boundary also has symmetry about the axes. In addition, the area enclosed by the rectangular cycle can represent the net power output (detailed below). Therefore, the *C*-*P* diagram can express the symmetry of Brayton cycle and conduct effective symmetry analysis. Therefore, the analysis method for Brayton cycles based on the proposed *C*-*P* diagram is noted as a kind of symmetry analysis method in this study.

As shown in Fig. 1b, Brayton cycle is a rectangle rotating counterclockwise in the *C*-*P* diagram, which is different from that in the conventional *T*-*s* diagram. For example, 1-2”−3”−4”, 1-2-3-4, and 1-2’−3’−4’ are three Brayton cycles starting from point 1, respectively. It can be seen that in the cycle 1-2”−3”−4”, the thermodynamic processes 1-2” and 3”−4” have a strong reflection symmetry relationship (the corresponding thermodynamic parameter points are close), while the rotational symmetry is weak (the difference between the rotated thermodynamic-cycle diagram and the original thermodynamic-cycle diagram are large). The rotational symmetry of cycle 1-2-3-4 is strong (rotating the rectangle 1-2-3-4 by n × 90 degrees coincides with itself), but its reflection symmetry is weak, because the corresponding parameters of its symmetric thermodynamic process (1-2 and 3-4) are quite different compared with that in 1-2”−3”−4”. According to the above analysis, 1-2’−3’−4’ is also a cycle with strong reflection symmetry and weak rotational symmetry. In the *C*-*P* diagram. The x-axial represents the pressure change in a compressor/expander, which is related to work transfer. Therefore, the lateral movement of a point in the diagram represents work transfer. Similarly, the longitudinal movement of a point represents heat transfer since the y-axial represents the temperature change of the working medium inside a heat exchanger.

Therefore, the net work of the Brayton cycle can be calculated by the product of constant *c*_*p*_ and the rectangular area surrounded by the cycle in Fig. 1b (the heat capacity of the working medium is a constant value since the ideal gas is considered in this section). In other words, the area of the Brayton cycle in the *C*-*P* diagram characterizes its network.

The following further unveils the key characteristics of the Brayton cycle using the proposed symmetry analysis method:

#### Intermediate temperature

Since point 1 is on the bisector and the isotherm is symmetric about the bisector, when the other two vertexes of the Brayton cycle lie on an isotherm line (i.e., the intermediate temperatures equals to each other), the system output work is maximum. For example, in Fig. 1b, when points 4 and 2 in cycle 1-2-3-4 are in the isotherm line *T*_m_ (*T*_m_ = *T*_2_ = *T*_4_), which are also symmetric about the bisector, the system output work is maximum because of the largest possible area enclosed by 1-2-3-4.

The coordinates of point 2 are ($\sqrt{{T}_{3}}$,$\sqrt{{T}_{1}}$). According to the relationship between the isotherm and the x-, y-axial variables, there is

4 $${{T}}_{{{{{{\rm{m}}}}}}}=\sqrt{{{T}}_{3}{{T}}_{1}}$$

The derived *T*_m_ in this study is the same as that reported in ref. ^10^. Clearly, this study demonstrates a much simpler approach to obtain this key characteristic information through the proposed symmetry analysis method compared to the prior study.

#### Output work

As aforementioned, it is easy to know that the work is the area surrounded by the cycle multiplied by *c*_p_. Since the coordinates of point 2 are ($\sqrt{{T}_{3}}$,$\sqrt{{T}_{1}}$), and the coordinates of point 1 are ($\sqrt{{T}_{1}}$,$\sqrt{{T}_{1}}$), the length of 1-2 is $\sqrt{{T}_{3}}-\sqrt{{T}_{1}}$. Then, the maximum work generated by this cycle is

5 $${{W}}_{{{{{{\rm{BR}}}}}},{{{{{\rm{MW}}}}}}}={{c}}_{{{{{{\rm{p}}}}}}}{\left(\sqrt{{{T}}_{{{{{{\rm{h}}}}}}}}-\sqrt{{{T}}_{{{{{{\rm{c}}}}}}}}\right)}^{2}={{c}}_{{{{{{\rm{p}}}}}}}\left({{T}}_{{{{{{\rm{h}}}}}}}+{{T}}_{{{{{{\rm{c}}}}}}}-2\sqrt{{{T}}_{{{{{{\rm{h}}}}}}}{{T}}_{{{{{{\rm{c}}}}}}}}\right)$$

#### System efficiency

For a general Brayton cycle without regenerative, considering the heat absorption area and using *C* to calculate the system efficiency, the following equation can be obtained:

6 $${\eta }_{{{{{{\rm{BR}}}}}}}=1-({{T}}_{{{{{{\rm{c}}}}}}}{{C}}_{{{{{{\rm{h}}}}}}})/({{T}}_{{{{{{\rm{h}}}}}}}{{C}}_{{{{{{\rm{c}}}}}}})$$

Since *C*_h_ > *C*_c_, it can be seen that the efficiency is always less than that of Carnot efficiency. The closer *C*_h_ to *C*_c_ is (i.e., in the *C*-*P* diagram the closer the rectangular upper line to the lower line is), the more efficient the system is.

**Fig. 2:**
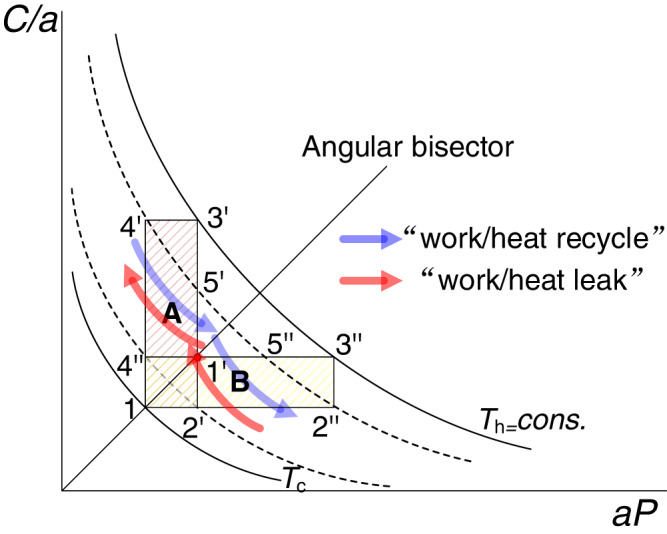
Increase reflection symmetry to increase system conversion efficiency. Area A represents the output work of cycle 1-2’−3’−4’. Area B represents the output work of cycle 1-2”−3”−4”. *T*_h_ and *T*_c_ are the upper and lower temperature bounds of the cycle, respectively. When heat/work flows from the exothermic/output-power end of the original system to the endothermic / input-power end, it is called work/heat recycle, which is helpful to increasing efficiency. When heat/work flows from the endothermic/input-power end of the original system to the exothermic/output-power end, it is called work/heat leak, which is bad for increasing efficiency.

When the Brayton cycle has heat recovery (as shown in cycle 1-2’−3’−4’ in Fig. 2), after deduction, the system efficiency under sufficient heat recovery is 1-*C*_c_/C_h_. According to the geometric constraints (see Figs. 1 and 2), ${C}_{{{{{{\rm{c}}}}}}}\sqrt{{T}_{{{{{{\rm{h}}}}}}}}/\sqrt{{T}_{{{{{{\rm{c}}}}}}}} < {C}_{{{{{{\rm{h}}}}}}} < {C}_{{{{{{\rm{c}}}}}}}{T}_{{{{{{\rm{h}}}}}}}/{T}_{{{{{{\rm{c}}}}}}}$ is satisfied for sufficient heat recovery. Therefore, the upper efficiency limit of this case is still Carnot efficiency, and then the endothermic process (2’- 3’) and exothermic process (4’- 1) are extreme symmetrical.

Meanwhile, Eq. (6) can be transformed into Eq. (7) for producing maximum work, according to the symmetrical geometric relationship, ${C}_{{{{{{\rm{h}}}}}}}=a\sqrt{{T}_{{{{{{\rm{h}}}}}}}}$ and ${C}_{{{{{{\rm{c}}}}}}}=a\sqrt{{T}_{{{{{{\rm{c}}}}}}}}$.

7 $${\eta }_{{{{{{\rm{BR}}}}}},{{{{{\rm{MW}}}}}}}=1-\sqrt{{T}_{{{{{{\rm{c}}}}}}}}/\sqrt{{T}_{{{{{{\rm{h}}}}}}}}$$

#### Terminal temperature and pressure

Since the initial point 1 and terminal point 3 are in the angular bisector, then:

8 $${a}^{2}={C}_{{{{{{\rm{h}}}}}}}/{P}_{3}={C}_{{{{{{\rm{c}}}}}}}/{P}_{1}$$

Substitute *C* into Eq. (8), the relationship between *T*_h_ and *T*_c_ is obtained:

9 $${T}_{{{{{{\rm{h}}}}}}}/{T}_{{{{{{\rm{c}}}}}}}={P}_{3}^{2}/{P}_{1}^{2}={C}_{{{{{{\rm{h}}}}}}}^{2}/{C}_{{{{{{\rm{c}}}}}}}^{2}$$

Furthermore, the power output of the system is maximum under strong rotational symmetry (rotating the rectangle 1−2−3−4 by n×90 degrees coincides with itself) but weak reflection symmetry (the distance 3 - 2 and 3 - 1 is far), the efficiency is the lowest. When enhancing the reflection symmetry of a Bryton cycle, e.g., changing 1-2−3−4 in Fig. 1b to 1–2”-3”-4”, the rotational symmetry is weakened. However, the cycle thermal-power conversion efficiency is improved as shown in Eq. (6) (when *T*_c_ and *T*_h_ is constant). It can be explained as follows. As shown in Fig. 2, when the reflection symmetry is enhanced, there is a coincidence of temperature zones (5”-4” and 2’ - 2”) to transfer the compression work of 2’- 2” to the expansion work of 5”-4”. We call this part of work as work recycle (see Fig. 2). From the perspective of circulation, this part of work is transferred without generating the network of circulation, so the power output is decreased. Furthermore, in the aspect of efficiency, its beneficial effect is to shift the heat absorption process to the larger *aP* and mean heat absorption temperature, which is closer to *T*_h_. At the same time, the average exothermic temperature is closer to *T*_c_ for being closer to 4”, then the efficiency is improved.

In cycle 1-2’−3’−4’, there is a heat transfer from process 4”-4’ to 2’-5’ (heat recycle). In addition, cycle 1-2’−3’−4’ and cycle 1-2”-3”-4” are symmetric to each other about the angular bisector. Thus, these two cycles have the same work output. Meanwhile, the absorbed heat of the two cycles are both from *T*_5’_ to *T*_3’_(*T*_5’_ = *T*_2”_; *T*_3’_ = *T*_3”_), thus the system efficiencies of the two cycles are also the same. Therefore, adopting heat recycle improves the system efficiency.

At the same time, when regenerative heating is not used for cycle 1-2’-3’-4’, the absorbed heat increases a lot compared with cycle 1-2”−3”−4”, whereas the work output remains unchanged. Under this circumstance, heat leak occurs and the system efficiency will be reduced (see Eq. (6)). Since the increase of symmetry is caused by heat leak, the efficiency does not improve.

Additionally, it’s worth noting that, when the cycle is for converting work to heat, the analysis process is similar to the above process. But at that condition, the product pursued is no longer the work represented by area enclosed by the rectangular cycle, but the heat represented by the area enclosed by the heat absorption / release process and the longitudinal axis, and the work consumed by the cycle is also the area enclosed by a rectangular cycle. Based on the above symmetry analysis approach, the corresponding conclusions can also be obtained.

### Effects of physical properties of working medium on the symmetry analysis of Brayton cycle

The above section demonstrates the proposed symmetry analysis method for Brayton cycle based on simplified working medium physical properties (e.g., constant *c*_p_) and initial state for the ease of understanding. In this section, the effects of the physical properties and initial states are further discussed in order to unveil the Brayton cycle in a more realistic setting.

First, when the *c*_p_ of the working medium is not a constant value, the work cannot be directly expressed through the enclosed area in Fig. 2b. Whereas we need to disassemble the Brayton cycle into several virtual cycles for analysis. The disassembly process is presented as follows.

Since *c*_p_ is a function of *T*, its function form *f* (*T*) and integral form *g* (*T*) are derived. The cycle 1-2”−3”−4” in Fig. 2 is taken as an example for work analysis, and the total work output of the system is

10 $${W}_{{{{{{\rm{BR}}}}}}(1-2{{\hbox{'}}}{{\hbox{'}}}-3{{\hbox{'}}}{{\hbox{'}}}-4{{\hbox{'}}}{{\hbox{'}}})}= 	{\int }_{{\!\!\!T}_{2{{\hbox{'}}}{{\hbox{'}}}}}^{{T}_{{{{{{\rm{h}}}}}}}}f(T)dT-{\int }_{{\!\!\!T}_{c}}^{{T}_{4{{\hbox{'}}}{{\hbox{'}}}}}f(T)dT\,\\ = 	\,g({T}_{{{{{{\rm{h}}}}}}})+g({T}_{{{{{{\rm{c}}}}}}})-(g({T}_{2{{\hbox{'}}}{{\hbox{'}}}})+g({T}_{4{{\hbox{'}}}{{\hbox{'}}}}))\,$$

When *g*(*x*) can be decomposed into *N* terms, and each term is monotone function of *K*_*i*_(*x*), then above equation can be evolved into:

11 $${W}_{{{{{{\rm{BR}}}}}}(1-2{{\hbox{'}}}{{\hbox{'}}}-3{{\hbox{'}}}{{\hbox{'}}}-4{{\hbox{'}}}{{\hbox{'}}})}=\mathop{\sum }\limits_{i}^{N}\left({K}_{i}\left({T}_{{{{{{\rm{h}}}}}}}\right)+{K}_{i}\left({T}_{{{{{{\rm{c}}}}}}}\right)-\left({K}_{i}\left({T}_{2{{\hbox{'}}}{{\hbox{'}}}}\right)+{K}_{i}\left({T}_{4{{\hbox{'}}}{{\hbox{'}}}}\right)\right)\right)$$

This way, the actual cycle is converted into *N* virtual cycles (*N* is any integer that is greater than 0). The *i*-th virtual cycle can be seen as a Brayton cycle with *C*_p_ = 1 kJ kg^−1^ K^−1^ in Fig. 2, and the temperatures of the four rectangular nodes are *K*_*i*_(*T*_h_), *K*_*i*_(*T*_c_), *K*_*i*_(*T*_2”_) and *K*_*i*_(*T*_4”_), respectively. The upper limit temperature and lower limit temperature are *K*_*i*_(*T*_h_) and *K*_*i*_(*T*_c_), respectively. Then the intermediate temperature corresponds to the maximum output power of the virtual cycle is ${K}_{i}({T}_{2{{\hbox{'}}}{{\hbox{'}}}})={K}_{i}({T}_{4{{\hbox{'}}}{{\hbox{'}}}})=\sqrt{{K}_{i}({T}_{{{{{{\rm{h}}}}}}}){K}_{i}({T}_{{{{{{\rm{c}}}}}}})}$, which is obtained by Eq. (4).

For any *i*, *K*_*i*_(*x*) has the symmetrical structure:

12 $${K}_{i}(\sqrt{{T}_{{{{{{\rm{h}}}}}}}{T}_{{{{{{\rm{c}}}}}}}})=\sqrt{{K}_{i}({T}_{{{{{{\rm{h}}}}}}}){K}_{i}({T}_{{{{{{\rm{c}}}}}}})}$$

Then ${T}_{2{{\hbox{'}}}{{\hbox{'}}}}={T}_{4{{\hbox{'}}}{{\hbox{'}}}}=\sqrt{{T}_{{{{{{\rm{h}}}}}}}{T}_{{{{{{\rm{c}}}}}}}}$ can make each virtual Brayton cycle output power maximum with a constant *C*_p_. Therefore, the actual Brayton cycle with a variable *C*_p_ also has the maximum output. In this way, *c*_p_ with functional form *f* (*T*) satisfying Eqs. (10–12) still makes the cycle symmetrical, and the maximum-work intermediate temperature is still$\sqrt{{T}_{{{{{{\rm{h}}}}}}}{T}_{{{{{{\rm{c}}}}}}}}$.

It should be noted that the conclusions of Eqs. (10–12) can be verified in many cases, for example, in refs. ^10,11^. As a further demonstration, the case where *c*_p_ is a linear function of temperature is presented in Supplementary Note 1.

In addition to the influence of *c*_p_ on Brayton cycle, working medium parameters such as *r* value and its initial state also affects the cycle performance. For example, when the working medium changes from air to carbon dioxide, the *r* value changes from 1.4 to 1.3, and the index ((*r*-1)/*r*) of *p* in *P* decreases from 0.2857 to 0.2308. According to Eq. (9), the pressure variation range corresponding to the maximum work increases within the same temperature range after *r* decreases.

When the inlet pressure of point 1 increases, *a* decrease and the range of *p* increases (see Eq. (9)). But, generally, the *P* range corresponding to the maximum work remains unchanged because the temperature variation is in a certain range.

When the inlet temperature of point 1 increases and *T*_h_ is constant, *a* increases and the range of *p* needs to be decreased for achieving maximum work. Because if the range of *p* remains unchanged, then the side length of the square will increase, which is inconsistent with the fact that the enclosed area should have decreased (the difference between outlet temperature and inlet temperature decreases).

### Universality of the relationship among symmetry, efficiency, and power

To convert thermal power in a thermodynamic plane with each point consisting of only two independent thermodynamic parameters (such as *T* and *p*), all processes of the thermodynamic cycle need to form a plane; and the formed plane consists of at least three processes. The realization of these three processes requires three types of thermal equipment. Correspondingly, if the thermal cycle is composed of four processes and each two processes correspond to each other, only two types of thermal equipment are required to realize the thermal cycle. In practice, this cycle composed of four basic processes is common, such as the Brayton cycle, Rankine cycle, Otto cycle, etc. Obviously, this kind of organizational cycle is also the cycle that theoretically requires the least types of equipment.

In addition to the analysis of Brayton cycle (see Sections “Results and Discussion” and “Conclusions”) using the symmetry method, in this section, a more common thermodynamic cycle with the following assumptions is analyzed: (1) the studied cycle consists of four processes; (2) the thermodynamic processes of the two interval processes are consistent; (3) The variation of *c*_p_ can be neglected.

**Fig. 3:**
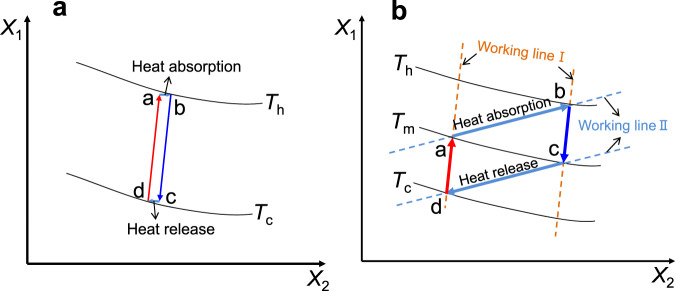
Relationship among the symmetry, efficiency and power. **a** Condition of strong reflection symmetry. *T*_h_ and *T*_c_ are the upper and lower temperature bounds of the cycle, respectively. a–d and b, c belong to the same type of equipment process, so they should be on a group of similar working lines; a, b and c, d belong to the same kind of equipment process and are on a group of working lines; *X*_1_ and *X*_2_ are thermodynamic parameters. **b** Condition of strong rotational symmetry. *T*_m_ is the maximum-work intermediate temperature of the cycle.

Figure 3 shows the relationship among the symmetry, efficiency and power of the above cycle. Here, the standard rectangular structure of the cycle diagram is not restricted. Figure 3a shows the beneficial effect of strong reflection symmetry on improving system efficiency. Due to the symmetry of process a-d and process b-c, the average heat-absorption temperature is close to the maximum temperature *T*_h_ (if a-d and b-c have heat exchange, the two processes can achieve deep regeneration), and the average heat release temperature is close to the minimum temperature *T*_c_. It can be seen that this strong reflection symmetry makes the system efficiency close to the ideal maximum efficiency, i.e., Carnot efficiency. However, this situation makes the heat absorption very small and the system power small. On the contrary, the case with strong rotational symmetry obtains the strongest heat absorption and power as shown in Fig. 3b because of the largest effective input heat (the temperature range in a-b that does not coincide with the temperature range of c-d is the largest). Otherwise, it indicates that there is heat leakage, as explained in Fig. 2.

In conclusion, the analysis of the above case proves the two conjectures that are listed in the Introduction section are correct under rational assumptions.

### Circular-shaped symmetrical thermodynamic cycle

**Fig. 4:**
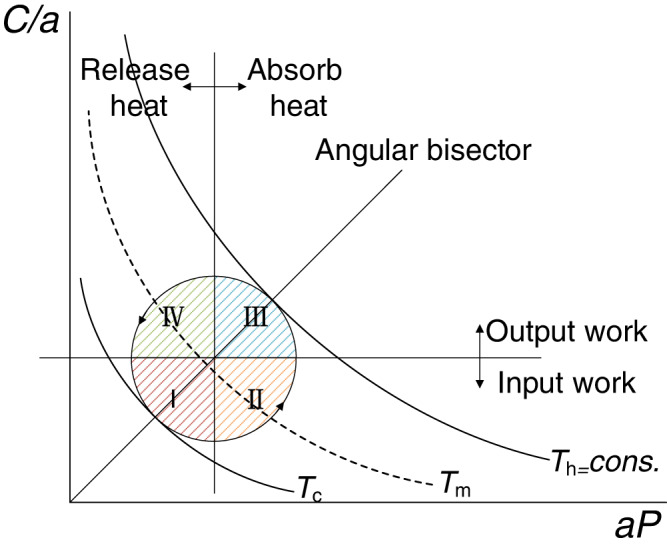
A more symmetrical cycle with circular-shaped structure. *T*_h_ and *T*_c_ are the upper and lower temperature bounds of the cycle, respectively. *T*_m_ is the intermediate temperature. Zone I: input work and release heat; Zone II: input work and absorb heat; Zone III: output work and absorb heat; Zone IV: output work and release heat.

Based on the above results, we propose herein a circular-shaped symmetrical thermodynamic cycle (single-phase working medium and small change in *c*_p_) as shown in Fig. 4. This cycle improves both work output and system efficiency. In practice, the four processes of this cycle can be realized through the combination of compression/expansion process and heat exchange process.

It can be seen that the area enclosed by the circle in Fig. 4 represents the net work output of the cycle. According to the geometry, the diameter of the circle is$\sqrt{{T}_{{{{{{\rm{h}}}}}}}}-\sqrt{{T}_{{{{{{\rm{c}}}}}}}}$, then the work generated by this cycle is calculated as

13 $$W=({{{{{\rm{\pi }}}}}}/2){c}_{{{{{{\rm{p}}}}}}}{\left(\sqrt{{T}_{{{{{{\rm{h}}}}}}}}-\sqrt{{T}_{{{{{{\rm{c}}}}}}}}\right)}^{2}=({{{{{\rm{\pi }}}}}}/2){W}_{{{{{{\rm{BR}}}}}},{{{{{\rm{MW}}}}}}}$$

By comparing Eq. (13) to Eq. (5), we can conclude that the work of the circular cycle is π/ 2 times greater than the maximum work of a conventional Brayton cycle.

Then, the system efficiency is deduced as

14 $$\eta =\frac{\sqrt{{T}_{{{{{{\rm{h}}}}}}}}-\sqrt{{T}_{{{{{{\rm{c}}}}}}}}}{\sqrt{{T}_{{{{{{\rm{h}}}}}}}}(\frac{1}{2}+\frac{\sqrt{2}}{{{{{{\rm{\pi }}}}}}})-\sqrt{{T}_{{{{{{\rm{c}}}}}}}}(\frac{1}{2}-\frac{\sqrt{2}}{{{{{{\rm{\pi }}}}}}})}$$

Comparing this efficiency with *η*_*BR,MW*_ in Eq. (7), it is obtained that

15 $$\frac{{\eta }_{{{{{{\rm{BR}}}}}},{{{{{\rm{MW}}}}}}}}{\eta }=\frac{1}{2}+\frac{\sqrt{2}}{{{{{{\rm{\pi }}}}}}}-\left(\frac{1}{2}-\frac{\sqrt{2}}{{{{{\pi }}}}}\right)(1-{\eta }_{{{{{{\rm{BR}}}}}},{{{{{\rm{MW}}}}}}})$$

It leads to the conclusion that *η* > *η*_BR,MW._ This is because under the same condition of reflection symmetry, the circular cycle has stronger rotational symmetry, which increases its work output and efficiency.

In addition to the internal circular cycle, the isotherms can also be internally connected with cycles composed of other symmetrical shapes. The following proves that when the number of sides increases, the power output and system efficiency are both improved. Here, we only consider the cycle with more than four sides. It can be seen that when the number of sides is greater than 4, the power output of the system increases, and the system efficiency can be expressed as:

16 $$\eta =\frac{\sqrt{{T}_{{{{{{\rm{h}}}}}}}}-\sqrt{{T}_{{{{{{\rm{c}}}}}}}}}{\theta \sqrt{{T}_{{{{{{\rm{h}}}}}}}}-(1-\theta )\sqrt{{T}_{{{{{{\rm{c}}}}}}}}}$$

where *θ* is a variable related to the number of cycle sides. It is easy to know that $(1/2+\sqrt{2}/\pi )\le \theta \le 1$. When the number of inner cycle edges increases, the efficiency is improved because *θ* tends to near lower boundary.

### Extending the symmetry analysis method to general energy conversion systems

Based on the above demonstration of using symmetry analysis method on Brayton cycle, this section further extends this symmetry method to general energy conversion systems.

For example, a general P-V-T system (each thermodynamic sub-process is a polytropic process with constant *pv*^*n*^) can also be illustrated as a cycle diagram with a rectangular structure (similar to the *C*-*P* diagram of Brayton cycle). The two working lines of this system type follow the equations of *pv*^*n*^ = *K*_1_ and *pv*^*m*^ = *K*_2_ (no isothermal process is involved, neither *m* nor *n* is equal to 1). Then, the x- and y-axial of the cycle diagram can be set to (*pv*^*n*^)^*x*^ and (*pv*^*m*^)^*y*^, respectively. Given that the ideal gas equation, in order to make the product of the *x*- and *y*-axial coordinates equal to *T*, the *x* and *y* need to satisfy Eq. (17).

17 $$\frac{x}{y}=\frac{m-1}{1-n}$$

The two coordinate parameters can be derived directly by using *pv*^*k*^ = *K*_1_ and *v* = *K*_2_. Then the abscissa of the established analysis graph is *v*^1-*k*^, while the ordinate is *pv*^*k*^. Specifically, for Otto cycle, since *m* or *n* is infinite, the x- and y- coordinates should be deduced separately rather than using Eq. (17).

For the symmetry analysis diagram established in this way, the analysis idea of Brayton cycle is still applicable. The area bounded by all process curves still represents the power, which is not limited to the assumption that zero work in the heat transfer process and the insulation in the work transfer process. Supplementary Note 2 and Supplementary Fig. S1 show the proof process.

**Fig. 5:**
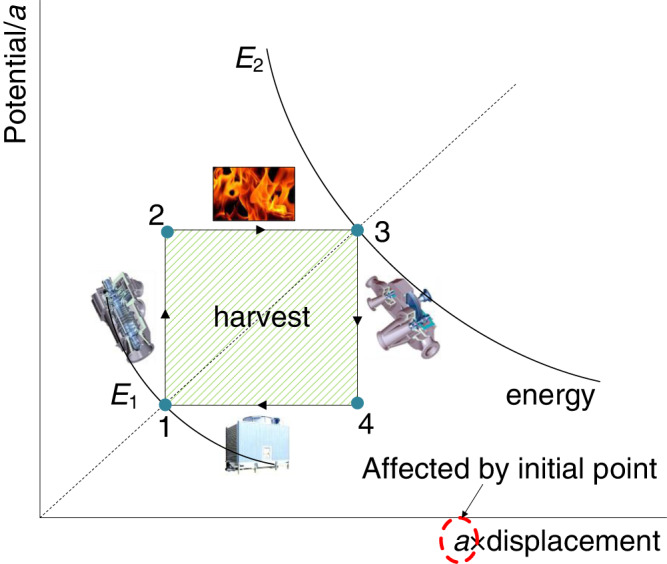
Potential-displacement-energy (PDE) diagram for energy conversion system analysis. *E*_1_ and *E*_2_ are the upper and lower energy bounds of the cycle, respectively. Points 1, 2, 3, and 4 are for the inlet of different device for different process, respectively. The selection of the value of *a* in the axial makes point 1 on the angle bisector line.

Besides Brayton cycle and other P-V-T cycles, general energy conversion systems can always be decomposed or transformed into the cycle process as shown in Fig. 5, in which process 1-2 and process 3-4 are the processes of potential increase and potential decrease, respectively (corresponding to the processes of changing Brayton cycle’s a*P* in Fig. 1), and 2–3 and 4–1 are the energy conversion processes on the equipotential line (corresponding to the processes of changing Brayton cycle’s *C/a* in Fig. 1). Due to the difference of 2−3 and 4-1 potentials, although the displacement is same, the exchanged energy is different, which results in net energy harvest as shown in the shadow area in Fig. 5. According to the previous analysis, the maximum benefit is achieved when the cycle 1-2-3−4 is a square (rotational symmetry is the strongest), and the system efficiency is also the lowest when energy recycling is considered. When the reflection symmetry is enhanced, the system energy conversion efficiency can be improved.

In order to draw the potential-displacement-energy (PDE) diagram, two pairs of quantitative processes need to be determined, and the product of the two quantities is the common boundary “energy”. In this way, the system working cycle can be expressed as a rectangular structure, and the energy boundary curve is symmetrical relative to the coordinate axes. Meanwhile, the area enclosed by the rectangle can represent the output product, then this method is very conducive to symmetry analysis and system optimization. Furthermore, some new cycles can be derived according to this principle.

For example, *T*-*s* diagram of Carnot cycle is a PDE diagram. *TS* is an energy used to describe the Carnot cycle. *TS* is called binding energy in thermodynamics (the part of energy that cannot be converted into work in the enthalpy with the isothermal isobaric chemical process), and Carnot cycle can be seen as a conversion process of this binding energy. The efficiency corresponding to the maximum work is $1-\sqrt{{(TS)}_{1}}/\sqrt{{(TS)}_{2}}$, in which (*TS*)_1_, (*TS*)_2_ are the lower boundary and the upper boundary of energy *TS*. Different from Brayton cycle, the input-heat process of this cycle changes the location into the upper equipotential line, while the input-heat process of Brayton cycle is in potential lifting process. Thus, the expression of system efficiency needs to change with temperature and entropy.

It can be seen that it has restriction for an energy conversion cycle preparing be drawn as a PDE diagram. The restriction is that the independent variables of the processes point are two (*x*_1_, *x*_2_), with the two independent variables being constant variables and satisfying the following equation:

18 $$f({x}_{1},{x}_{2})=\left\{\begin{array}{c}g({x}_{1})y({x}_{2})=h\hfill \\ g({x}_{i},{x}_{k})y({x}_{j})=h \,\, \qquad i,k \, \ne \, 1;j=2\hfill \\ g({x}_{i},{x}_{k})y({x}_{j},{x}_{l})=h\quad\,i,k \, \ne \, 1;\,j,l\ne 2\,\end{array}\right.$$

where *x*_***i***_ is a thermodynamic state parameter (temperature, pressure, specific volume, entropy, specific enthalpy, specific internal energy) or its calculated parameter. *h* is energy. In Eq. (18), the invariants of *x*_1_ and *x*_2_ are transformed into *g*-function and *y*-function. The product of *g*-function and *y*-function equals to energy.

In general, the proposed symmetry analysis method in this paper provides new insights on studying and understanding thermodynamic cycles thoroughly. Additionally, according to the proposed P-D-E diagram, new cycles with symmetry characteristics and better performance can be created and it’s easy to find the directions for system parameter optimization. Furthermore, the symmetry principle proposed in this paper can be applied for establishing and/or analyzing complex large-scale energy systems (complex large-scale energy system can be decomposed into several basic cycles), which will be studied in future works.

## Conclusions

In the past, symmetry analysis was only used for micro or abstract objects. In this paper, the symmetry concept is applied to analysis macro thermodynamic system, and the Brayton cycle is taken as an example. The proposed symmetry analysis method is further extended into the analysis of general energy conversion systems. The conclusions are as follows:

1. Ideal Brayton cycle shows the symmetric structure after parameters transformation. The maximum output power is achieved with the strongest rotational symmetry. The reflection symmetry of the system is enhanced with increasing system efficiency. Through the methodology, the optimal expressions of key parameters are easily obtained. When the integral of specific heat capacity (*c*_p_) to temperature can be disassembled into the symmetric structure of temperature along with constraints met, *c*_p_ has no effect on the intermediate maximum-work temperature.
2. The relationship between symmetry, efficiency and power is obtained. Meanwhile, according to this relationship, a more symmetrical circular cycle shown in the *C*-*P* diagram is proposed and studied. The cycle is also in the physical property range of the working medium of Brayton cycle (single-phase, small change in *c*_p_), and consists of traditional compression/expansion and heat exchange processes. The work output of this cycle is π/2 times greater than that of Brayton cycle, and the system efficiency is also improved.
3. The symmetry principle can be used to analyze general energy conversion systems with the proposed potential-displacement-energy (PDE) diagram. Generally, the energy conversion process can be divided into two potential-change and two equipotential processes. Running the same displacement on different potentials can exchange different energy, and then harvest benefits. General P-V-T systems can be easily drawn into PDE diagram for analysis. *TS* can be used as the boundary energy corresponding to Carnot cycle analysis.

### Supplementary information

Supplementary Information

## Data Availability

The data that support the findings of this study are available from the corresponding author upon request.
