# Supplementary material for: A symmetry analysis methodology for general energy conversion systems
Source: Commun Eng. 2023 Jul 25;2:49. doi: 10.1038/s44172-023-00096-x (PMC10955881; doi:10.1038/s44172-023-00096-x)
Supplement: Supplementary file 1 — Supplementary Information [file 44172_2023_96_MOESM1_ESM.pdf]

Supplementary Note 1. Symmetry of working fluid properties in Brayton cycle

When the expression of specific heat capacity is $cT+d$, the output work of Brayton cycle is calculated as:

$$\begin{aligned} W &= \int_{T_{2''}}^{T_{3''}} (cT+d)dT - \int_{T_1}^{T_{4''}} (cT+d)dT \\ &= \frac{c}{2}\left[T_\mathrm{h}^2 + T_\mathrm{c}^2 - \left(T_{2''}^2 + T_{4''}^2\right)\right] + d\left[T_\mathrm{h} + T_\mathrm{c} - (T_{2''} + T_{4''})\right] \end{aligned} \tag{S1}$$

In eq. (S1), it can be seen that for the second square bracket, when $T_{2''} = T_{4''}$, the value in the bracket can be maximized. Similarly, for the first square bracket, $T_{2''}^2 = T_{4''}^2 = \sqrt{T_{2''}^2 T_{4''}^2}$ corresponding to $T_{2''} = T_{4''} = \sqrt{T_{2''} T_{4''}}$ can lead the value in the bracket largest. Thereby, the optimum intermediate temperature corresponding to the maximum value of the two parts is the same. Therefore, regardless of the relative size relationship and sign relationship between c and d, the intermediate temperature is determined.

Similarly, this conclusion is also valid when the specific heat capacity appears in the form of other polynomials and power equations.

Supplementary Note 2. Relationship between process surrounding area and net output power in symmetry analysis diagram of P-V-T system

Fig. S1 is the symmetry analysis diagram of general P-V-T systems. As stated in this paper, in order to make equipment corresponding and to reduce equipment type, the polytropic exponents of process T_1-T_2 and process T_3-T_4 are the same, both m; The polytropic exponents of process T_2-T_3 and process T_4-T_1 are also the same, both n.

Then the heat exchange of each process is:

$$\begin{cases} Q_1 = L(m)\cdot(T_2 - T_1) \\ Q_2 = L(n)\cdot(T_3 - T_2) \\ Q_3 = L(m)\cdot(T_4 - T_3) \\ Q_2 = L(n)\cdot(T_1 - T_4) \end{cases} \tag{S2}$$

where $L(x)$ is the function of polytropic exponent, shown in eq. (S3).

$$L(x) = \frac{x-k}{x-1} c_\mathrm{v} \tag{S3}$$

where c_v is specific heat at constant volume.

By summing the heat of each part of eq. (S2), the net output work can be obtained:

$$W = \sum Q_i = [L(m) - L(n)][(T_2 - T_1) - (T_3 - T_4)] \tag{S4}$$

In eq. (S4), $[(T_2 - T_1) - (T_3 - T_4)]$ is the area of the shaded part in Fig. S1. Therefore, the system output power is proportional to the area enclosed by processes, and the proportional coefficient is a function of two polytropic exponents.

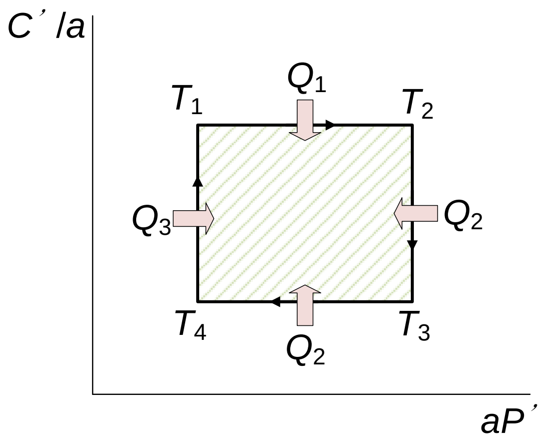

Fig. S1 The symmetry analysis diagram of P-V-T system. *C’* and *P’* are the parameters derived by eq. (17)). *Q* represent exchanged heat. T_1, T_2, T_3 and T_4 represent the temperature of different point, respectively.
